# 5-(4-Bromo­phen­oxy)-1-methyl-3-methyl-1*H*-pyrazole-4-carbaldehyde-*O*-[(5-meth­oxy-1,3,4-thia­diazol-2-yl)-meth­yl]oxime

**DOI:** 10.1107/S1600536812042274

**Published:** 2012-10-13

**Authors:** Chong-Guang Fan, Jian-Cun Chen, Hong Dai, Yun-Hua Wei, Yu-Jun Shi

**Affiliations:** a College of Chemistry and Chemical Engineering, Nantong University, Nantong 226019, Peoples’ Republic of China

## Abstract

In the title mol­ecule, C_16_H_16_BrN_5_O_3_S, the 1,3,4-thia­diazole ring is situated under the benzene ring, forming a dihedral angle of 86.6 (2)°, and with an S⋯*Cg* (where *Cg* is the centroid of the benzene ring) distance of 3.312 (3) Å. The benzene and 1,3,4-thia­diazole rings form dihedral angles of 83.8 (3) and 57.7 (2)°, respectively, with the central pyrazole ring. In the absence of classical hydrogen bonds, the crystal packing is stabilized by a C—H⋯π inter­action..

## Related literature

For a related structure, see: Dai *et al.* (2011 ▶).
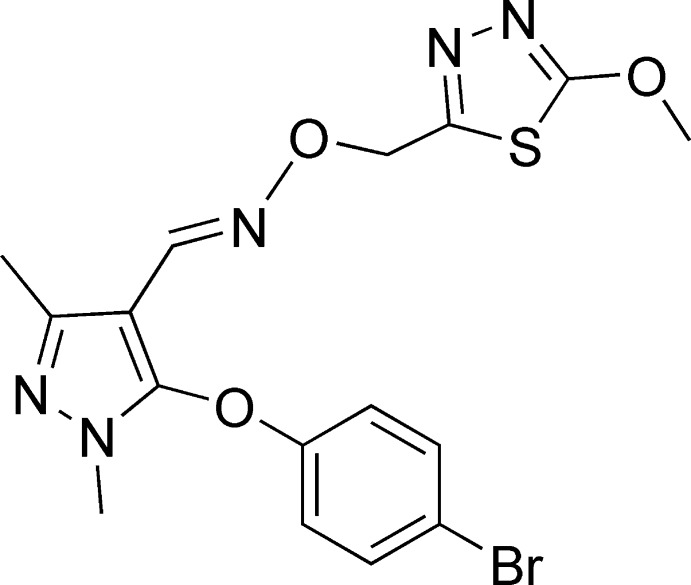


## Experimental

### 

#### Crystal data

C_16_H_16_BrN_5_O_3_S
*M* *_r_* = 438.31
Triclinic, 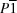

*a* = 9.732 (3) Å
*b* = 9.832 (2) Å
*c* = 11.166 (3) Å
α = 64.55 (2)°
β = 69.62 (2)°
γ = 75.33 (3)°
*V* = 897.5 (4) Å^3^
*Z* = 2
Mo *K*α radiation
μ = 2.43 mm^−1^
*T* = 113 K
0.20 × 0.18 × 0.12 mm

#### Data collection

Rigaku Saturn724 CCD diffractometer
Absorption correction: multi-scan (*CrystalClear*; Rigaku, 2008 ▶) *T*_min_ = 0.642, *T*_max_ = 0.759
7729 measured reflections
3175 independent reflections
2363 reflections with *I* > 2σ(*I*)
*R*_int_ = 0.044

#### Refinement

*R*[*F* ^2^ > 2σ(*F* ^2^)] = 0.029
*wR*(*F* ^2^) = 0.054
*S* = 1.02
3175 reflections
238 parameters
H-atom parameters constrained
Δρ_max_ = 0.36 e Å^−3^
Δρ_min_ = −0.49 e Å^−3^

### 

Data collection: *CrystalClear* (Rigaku, 2008 ▶); cell refinement: *CrystalClear*; data reduction: *CrystalClear*; program(s) used to solve structure: *SHELXS97* (Sheldrick, 2008 ▶); program(s) used to refine structure: *SHELXL97* (Sheldrick, 2008 ▶); molecular graphics: *SHELXTL* (Sheldrick, 2008 ▶); software used to prepare material for publication: *SHELXTL*.

## Supplementary Material

Crystal structure: contains datablock(s) global, I. DOI: 10.1107/S1600536812042274/cv5348sup1.cif

Structure factors: contains datablock(s) I. DOI: 10.1107/S1600536812042274/cv5348Isup2.hkl

Supplementary material file. DOI: 10.1107/S1600536812042274/cv5348Isup3.cml

Additional supplementary materials:  crystallographic information; 3D view; checkCIF report

## Figures and Tables

**Table 1:** Hydrogen-bond geometry (Å, °) *Cg* is the centroid of the C1–C6 ring.

*D*—H⋯*A*	*D*—H	H⋯*A*	*D*⋯*A*	*D*—H⋯*A*
C11—H11*A*⋯*Cg* ^i^	0.98	2.89	3.652 (4)	125

Symmetry code: (i) 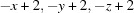.

**Fig. 1.**
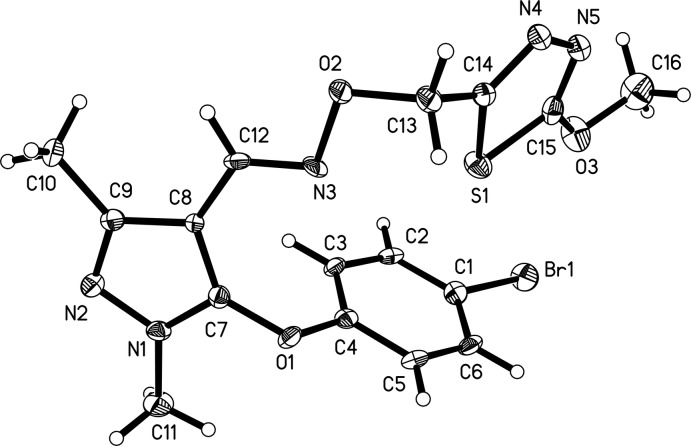
The molecular structure of (I) showing the atomic numbering and 50% probabilty displacement ellipsoids.

## supplementary crystallographic information

### Comment

In a continuation of our structural study of pyrazole oxime derivatives (Dai *et al.*, 2011), we report here the crystal structure of the title compound, (I). In (I) (Fig. 1), all bonds lengths and angles are similar to those observed in the related compound (Dai *et al.*, 2011). The dihedral angles between the substituted phenyl ring and the pyrazole ring and between the 1,3,4-thiadiazole ring and the pyrazole ring are 83.8 (3)° and 57.7 (2)°, respectively. The crystal packing displays short intermolecular C···C contacts of 3.203 (4) Å.

### Experimental

To a stirred solution of 1-methyl-3-methyl-5-(4-bromophenoxy)-1*H*-pyrazole -4-carbaldehyde oxime (3 mmol), and powdered potassium carbonate (9 mmol) in 30 ml of anhydrous acetonitrile, was added 2-chloromethyl-5-methoxy- 1,3,4-thiadiazole (4.2 mmol) at room temperature. The mixture was heated to reflux for 13 h. After removal of the solvent, the residue was separated by column chromatography on silica gel using a mixture of petroleum ether/ethyl acetate to obtain colourless crystals.

### Refinement

All H atoms were placed in calculated positions, with C–H = 0.95–0.99 Å, and included in the final cycles of refinement using a riding model, with *U*_iso_(H) = 1.2*U*_eq_(C).

### Crystal data

**Table:**

C_16_H_16_BrN_5_O_3_S	*Z* = 2
*M**_r_* = 438.31	*F*(000) = 444
Triclinic, *P*1	*D*_x_ = 1.622 Mg m^−^^3^
Hall symbol: -P 1	Mo *K*α radiation, λ = 0.71073 Å
*a* = 9.732 (3) Å	Cell parameters from 3391 reflections
*b* = 9.832 (2) Å	θ = 2.1–27.9°
*c* = 11.166 (3) Å	µ = 2.43 mm^−^^1^
α = 64.55 (2)°	*T* = 113 K
β = 69.62 (2)°	Prism, colourless
γ = 75.33 (3)°	0.20 × 0.18 × 0.12 mm
*V* = 897.5 (4) Å^3^	

### Data collection

**Table:**

Rigaku Saturn724 CCD diffractometer	3175 independent reflections
Radiation source: rotating anode	2363 reflections with *I* > 2σ(*I*)
Multilayer monochromator	*R*_int_ = 0.044
Detector resolution: 14.22 pixels mm^-1^	θ_max_ = 25.0°, θ_min_ = 2.1°
ω and φ scans	*h* = −11→11
Absorption correction: multi-scan (*CrystalClear*; Rigaku, 2008)	*k* = −11→11
*T*_min_ = 0.642, *T*_max_ = 0.759	*l* = −13→13
7729 measured reflections	

### Refinement

**Table:**

Refinement on *F*^2^	Primary atom site location: structure-invariant direct methods
Least-squares matrix: full	Secondary atom site location: difference Fourier map
*R*[*F*^2^ > 2σ(*F*^2^)] = 0.029	Hydrogen site location: inferred from neighbouring sites
*wR*(*F*^2^) = 0.054	H-atom parameters constrained
*S* = 1.02	*w* = 1/[σ^2^(*F*_o_^2^) + (0.*P*)^2^] where *P* = (*F*_o_^2^ + 2*F*_c_^2^)/3
3175 reflections	(Δ/σ)_max_ = 0.001
238 parameters	Δρ_max_ = 0.36 e Å^−^^3^
0 restraints	Δρ_min_ = −0.49 e Å^−^^3^

### Special details

**Table:**

Geometry. All e.s.d.'s (except the e.s.d. in the dihedral angle between two l.s. planes) are estimated using the full covariance matrix. The cell e.s.d.'s are taken into account individually in the estimation of e.s.d.'s in distances, angles and torsion angles; correlations between e.s.d.'s in cell parameters are only used when they are defined by crystal symmetry. An approximate (isotropic) treatment of cell e.s.d.'s is used for estimating e.s.d.'s involving l.s. planes.
Refinement. Refinement of *F*^2^ against ALL reflections. The weighted *R*-factor *wR* and goodness of fit *S* are based on *F*^2^, conventional *R*-factors *R* are based on *F*, with *F* set to zero for negative *F*^2^. The threshold expression of *F*^2^ > σ(*F*^2^) is used only for calculating *R*-factors(gt) *etc*. and is not relevant to the choice of reflections for refinement. *R*-factors based on *F*^2^ are statistically about twice as large as those based on *F*, and *R*- factors based on ALL data will be even larger.

### Fractional atomic coordinates and isotropic or equivalent isotropic displacement parameters (Å^2^)

**Table:**

	*x*	*y*	*z*	*U*_iso_*/*U*_eq_	
Br1	0.51369 (3)	0.66558 (3)	1.32176 (3)	0.02651 (10)	
S1	0.54286 (8)	0.73735 (8)	0.87231 (7)	0.02150 (19)	
O1	0.96309 (18)	0.88211 (18)	0.76885 (17)	0.0166 (5)	
O2	0.63830 (19)	0.93453 (19)	0.53541 (18)	0.0202 (5)	
O3	0.3297 (2)	0.6561 (2)	1.10298 (18)	0.0291 (5)	
N1	1.0439 (2)	1.1205 (2)	0.6791 (2)	0.0157 (5)	
N2	1.0308 (2)	1.2623 (2)	0.5802 (2)	0.0173 (6)	
N3	0.7358 (2)	0.9392 (2)	0.6035 (2)	0.0166 (5)	
N4	0.3648 (2)	0.7120 (2)	0.7625 (2)	0.0196 (6)	
N5	0.2883 (2)	0.6764 (2)	0.9015 (2)	0.0202 (6)	
C1	0.6530 (3)	0.7382 (3)	1.1473 (3)	0.0174 (7)	
C2	0.6437 (3)	0.8902 (3)	1.0675 (3)	0.0161 (7)	
H2	0.5676	0.9587	1.0999	0.019*	
C3	0.7454 (3)	0.9447 (3)	0.9390 (3)	0.0155 (7)	
H3	0.7395	1.0497	0.8827	0.019*	
C4	0.8547 (3)	0.8423 (3)	0.8958 (3)	0.0143 (6)	
C5	0.8662 (3)	0.6893 (3)	0.9768 (3)	0.0168 (7)	
H5	0.9440	0.6213	0.9455	0.020*	
C6	0.7646 (3)	0.6357 (3)	1.1029 (3)	0.0176 (7)	
H6	0.7705	0.5305	1.1587	0.021*	
C7	0.9571 (3)	1.0319 (3)	0.6821 (3)	0.0144 (7)	
C8	0.8804 (3)	1.1148 (3)	0.5847 (3)	0.0114 (6)	
C9	0.9324 (3)	1.2589 (3)	0.5235 (3)	0.0150 (7)	
C10	0.8892 (3)	1.3946 (3)	0.4084 (2)	0.0211 (7)	
H10A	0.9228	1.4843	0.4016	0.032*	
H10B	0.7816	1.4105	0.4264	0.032*	
H10C	0.9350	1.3784	0.3216	0.032*	
C11	1.1437 (3)	1.0799 (3)	0.7631 (3)	0.0239 (7)	
H11A	1.1112	1.1413	0.8199	0.036*	
H11B	1.2437	1.0986	0.7032	0.036*	
H11C	1.1436	0.9721	0.8229	0.036*	
C12	0.7799 (3)	1.0706 (3)	0.5427 (3)	0.0153 (6)	
H12	0.7448	1.1421	0.4664	0.018*	
C13	0.5995 (3)	0.7852 (3)	0.5917 (3)	0.0194 (7)	
H13A	0.6904	0.7119	0.5950	0.023*	
H13B	0.5533	0.7753	0.5301	0.023*	
C14	0.4958 (3)	0.7459 (3)	0.7328 (3)	0.0151 (6)	
C15	0.3691 (3)	0.6834 (3)	0.9682 (3)	0.0194 (7)	
C16	0.1869 (3)	0.6019 (3)	1.1781 (3)	0.0360 (9)	
H16A	0.1105	0.6772	1.1391	0.054*	
H16B	0.1657	0.5860	1.2754	0.054*	
H16C	0.1879	0.5058	1.1708	0.054*	

### Atomic displacement parameters (Å^2^)

**Table:**

	*U*^11^	*U*^22^	*U*^33^	*U*^12^	*U*^13^	*U*^23^
Br1	0.02695 (19)	0.03281 (19)	0.01571 (17)	−0.00625 (14)	−0.00270 (14)	−0.00650 (15)
S1	0.0216 (4)	0.0264 (4)	0.0212 (4)	−0.0059 (4)	−0.0073 (3)	−0.0105 (4)
O1	0.0170 (11)	0.0132 (10)	0.0140 (11)	0.0018 (8)	−0.0045 (8)	−0.0019 (9)
O2	0.0230 (12)	0.0190 (11)	0.0215 (11)	−0.0089 (9)	−0.0136 (9)	−0.0009 (10)
O3	0.0335 (13)	0.0366 (13)	0.0201 (12)	−0.0128 (11)	−0.0003 (10)	−0.0140 (11)
N1	0.0149 (14)	0.0174 (13)	0.0178 (13)	−0.0006 (11)	−0.0085 (11)	−0.0070 (11)
N2	0.0196 (14)	0.0154 (13)	0.0160 (13)	−0.0016 (11)	−0.0063 (11)	−0.0044 (11)
N3	0.0142 (13)	0.0213 (13)	0.0184 (13)	−0.0059 (11)	−0.0091 (11)	−0.0056 (12)
N4	0.0185 (14)	0.0196 (13)	0.0202 (14)	−0.0064 (11)	−0.0067 (11)	−0.0036 (12)
N5	0.0190 (15)	0.0195 (14)	0.0211 (14)	−0.0057 (11)	−0.0055 (11)	−0.0050 (12)
C1	0.0212 (17)	0.0228 (16)	0.0088 (15)	−0.0060 (14)	−0.0058 (13)	−0.0034 (14)
C2	0.0157 (16)	0.0167 (15)	0.0195 (17)	0.0050 (13)	−0.0105 (13)	−0.0095 (14)
C3	0.0175 (16)	0.0106 (14)	0.0180 (16)	0.0011 (13)	−0.0096 (13)	−0.0030 (13)
C4	0.0155 (16)	0.0176 (15)	0.0121 (15)	−0.0018 (13)	−0.0075 (12)	−0.0049 (13)
C5	0.0168 (16)	0.0161 (15)	0.0170 (16)	0.0052 (13)	−0.0087 (13)	−0.0066 (13)
C6	0.0220 (17)	0.0134 (15)	0.0184 (16)	0.0020 (13)	−0.0130 (13)	−0.0034 (14)
C7	0.0130 (16)	0.0143 (15)	0.0139 (15)	−0.0015 (13)	−0.0012 (12)	−0.0057 (13)
C8	0.0103 (16)	0.0103 (14)	0.0130 (15)	−0.0014 (12)	−0.0040 (12)	−0.0031 (13)
C9	0.0132 (16)	0.0184 (15)	0.0110 (15)	0.0002 (13)	−0.0023 (13)	−0.0054 (13)
C10	0.0225 (17)	0.0156 (15)	0.0202 (17)	−0.0059 (13)	−0.0059 (14)	−0.0003 (14)
C11	0.0274 (19)	0.0275 (18)	0.0245 (17)	−0.0033 (15)	−0.0169 (14)	−0.0091 (15)
C12	0.0100 (16)	0.0194 (16)	0.0137 (16)	0.0028 (13)	−0.0055 (12)	−0.0043 (14)
C13	0.0216 (17)	0.0196 (16)	0.0213 (17)	−0.0060 (14)	−0.0097 (14)	−0.0067 (14)
C14	0.0198 (17)	0.0106 (14)	0.0219 (17)	−0.0005 (13)	−0.0105 (13)	−0.0096 (13)
C15	0.0218 (18)	0.0133 (15)	0.0204 (17)	−0.0033 (13)	−0.0021 (14)	−0.0061 (14)
C16	0.034 (2)	0.039 (2)	0.0244 (19)	−0.0063 (17)	0.0040 (16)	−0.0103 (17)

### Geometric parameters (Å, º)

**Table:**

Br1—C1	1.897 (2)	C3—H3	0.9500
S1—C15	1.730 (3)	C4—C5	1.381 (3)
S1—C14	1.735 (3)	C5—C6	1.376 (3)
O1—C7	1.371 (3)	C5—H5	0.9500
O1—C4	1.402 (3)	C6—H6	0.9500
O2—C13	1.420 (3)	C7—C8	1.378 (3)
O2—N3	1.426 (3)	C8—C9	1.422 (3)
O3—C15	1.337 (3)	C8—C12	1.442 (3)
O3—C16	1.449 (3)	C9—C10	1.492 (3)
N1—C7	1.342 (3)	C10—H10A	0.9800
N1—N2	1.365 (3)	C10—H10B	0.9800
N1—C11	1.448 (3)	C10—H10C	0.9800
N2—C9	1.333 (3)	C11—H11A	0.9800
N3—C12	1.279 (3)	C11—H11B	0.9800
N4—C14	1.293 (3)	C11—H11C	0.9800
N4—N5	1.395 (3)	C12—H12	0.9500
N5—C15	1.287 (3)	C13—C14	1.489 (3)
C1—C2	1.370 (3)	C13—H13A	0.9900
C1—C6	1.393 (3)	C13—H13B	0.9900
C2—C3	1.395 (3)	C16—H16A	0.9800
C2—H2	0.9500	C16—H16B	0.9800
C3—C4	1.376 (3)	C16—H16C	0.9800
			
C15—S1—C14	85.62 (13)	N2—C9—C10	121.1 (2)
C7—O1—C4	117.5 (2)	C8—C9—C10	127.0 (3)
C13—O2—N3	109.17 (19)	C9—C10—H10A	109.5
C15—O3—C16	114.5 (2)	C9—C10—H10B	109.5
C7—N1—N2	111.0 (2)	H10A—C10—H10B	109.5
C7—N1—C11	127.8 (2)	C9—C10—H10C	109.5
N2—N1—C11	121.2 (2)	H10A—C10—H10C	109.5
C9—N2—N1	105.0 (2)	H10B—C10—H10C	109.5
C12—N3—O2	108.0 (2)	N1—C11—H11A	109.5
C14—N4—N5	113.1 (2)	N1—C11—H11B	109.5
C15—N5—N4	110.5 (2)	H11A—C11—H11B	109.5
C2—C1—C6	121.0 (2)	N1—C11—H11C	109.5
C2—C1—Br1	119.8 (2)	H11A—C11—H11C	109.5
C6—C1—Br1	119.2 (2)	H11B—C11—H11C	109.5
C1—C2—C3	120.2 (2)	N3—C12—C8	122.9 (3)
C1—C2—H2	119.9	N3—C12—H12	118.5
C3—C2—H2	119.9	C8—C12—H12	118.5
C4—C3—C2	118.3 (2)	O2—C13—C14	112.9 (2)
C4—C3—H3	120.8	O2—C13—H13A	109.0
C2—C3—H3	120.8	C14—C13—H13A	109.0
C3—C4—C5	121.7 (2)	O2—C13—H13B	109.0
C3—C4—O1	124.0 (2)	C14—C13—H13B	109.0
C5—C4—O1	114.3 (2)	H13A—C13—H13B	107.8
C6—C5—C4	119.8 (3)	N4—C14—C13	123.1 (2)
C6—C5—H5	120.1	N4—C14—S1	114.4 (2)
C4—C5—H5	120.1	C13—C14—S1	122.53 (19)
C5—C6—C1	118.9 (2)	N5—C15—O3	126.0 (2)
C5—C6—H6	120.5	N5—C15—S1	116.3 (2)
C1—C6—H6	120.5	O3—C15—S1	117.6 (2)
N1—C7—O1	119.3 (3)	O3—C16—H16A	109.5
N1—C7—C8	109.1 (2)	O3—C16—H16B	109.5
O1—C7—C8	131.5 (2)	H16A—C16—H16B	109.5
C7—C8—C9	103.0 (2)	O3—C16—H16C	109.5
C7—C8—C12	130.8 (2)	H16A—C16—H16C	109.5
C9—C8—C12	126.0 (3)	H16B—C16—H16C	109.5
N2—C9—C8	111.9 (2)		
			
C7—N1—N2—C9	0.7 (3)	N1—C7—C8—C12	176.5 (2)
C11—N1—N2—C9	179.4 (2)	O1—C7—C8—C12	0.4 (5)
C13—O2—N3—C12	174.10 (19)	N1—N2—C9—C8	−0.1 (3)
C14—N4—N5—C15	−1.2 (3)	N1—N2—C9—C10	−179.3 (2)
C6—C1—C2—C3	0.8 (4)	C7—C8—C9—N2	−0.5 (3)
Br1—C1—C2—C3	−179.91 (19)	C12—C8—C9—N2	−176.4 (2)
C1—C2—C3—C4	−0.4 (4)	C7—C8—C9—C10	178.7 (2)
C2—C3—C4—C5	−0.7 (4)	C12—C8—C9—C10	2.8 (4)
C2—C3—C4—O1	179.6 (2)	O2—N3—C12—C8	−179.9 (2)
C7—O1—C4—C3	−1.3 (4)	C7—C8—C12—N3	6.2 (4)
C7—O1—C4—C5	179.0 (2)	C9—C8—C12—N3	−179.1 (2)
C3—C4—C5—C6	1.4 (4)	N3—O2—C13—C14	72.8 (3)
O1—C4—C5—C6	−178.9 (2)	N5—N4—C14—C13	178.9 (2)
C4—C5—C6—C1	−1.1 (4)	N5—N4—C14—S1	0.4 (3)
C2—C1—C6—C5	0.0 (4)	O2—C13—C14—N4	118.3 (3)
Br1—C1—C6—C5	−179.35 (19)	O2—C13—C14—S1	−63.3 (3)
N2—N1—C7—O1	175.63 (19)	C15—S1—C14—N4	0.4 (2)
C11—N1—C7—O1	−3.0 (4)	C15—S1—C14—C13	−178.2 (2)
N2—N1—C7—C8	−1.0 (3)	N4—N5—C15—O3	179.9 (2)
C11—N1—C7—C8	−179.6 (2)	N4—N5—C15—S1	1.5 (3)
C4—O1—C7—N1	98.1 (3)	C16—O3—C15—N5	5.5 (4)
C4—O1—C7—C8	−86.2 (3)	C16—O3—C15—S1	−176.12 (18)
N1—C7—C8—C9	0.9 (3)	C14—S1—C15—N5	−1.1 (2)
O1—C7—C8—C9	−175.2 (2)	C14—S1—C15—O3	−179.7 (2)

### Hydrogen-bond geometry (Å, º)

Cg is the centroid of the C1–C6 ring.

**Table:**

*D*—H···*A*	*D*—H	H···*A*	*D*···*A*	*D*—H···*A*
C11—H11*A*···*Cg*^i^	0.98	2.89	3.652 (4)	125

Symmetry code: (i) −*x*+2, −*y*+2, −*z*+2.
